# The Constrained Disorder Principle: A Paradigm Shift for Accurate Interactome Mapping and Information Analysis in Complex Biological Systems

**DOI:** 10.3390/bioengineering12111255

**Published:** 2025-11-16

**Authors:** Yaron Ilan

**Affiliations:** Department of Medicine, Hadassah Medical Center, Faculty of Medicine, Hebrew University, Jerusalem 91120, Israel; ilan@hadassah.org.il

**Keywords:** interactome, constrained disorder principle, biological variability, network physiology, systems biology, molecular interactions, stochastic processes, dynamic networks

## Abstract

The interactome, which represents the comprehensive network of molecular interactions within biological systems, has become a crucial framework for understanding cellular functions and disease mechanisms. However, current interactome models face significant limitations because they fail to account for the inherent variability and randomness of biological systems. The Constrained Disorder Principle (CDP) offers an innovative approach to addressing these limitations by integrating physiological variability and biological noise as essential components rather than viewing them as experimental artifacts. This paper examines how the CDP may enhance the accuracy of interactome models by incorporating the dynamic and variable nature of biological systems while preserving functional constraints. We suggest that incorporating controlled variability into interactome models may significantly improve their predictive power and biological relevance. This shift moves away from static network representations toward dynamic, context-dependent interaction maps that more accurately reflect the reality of living systems. Through a comprehensive analysis of existing clinical data and theoretical frameworks, we propose methodological advances and provide evidence for the functional importance of biological variability at the molecular, cellular, and organ levels.

## 1. Introduction

An interactome is a detailed representation of the functional interactions between molecules, whether within a cell or throughout an entire organism. These interactomes often reveal significant interactions between molecules that may not initially appear to be functionally related [1,2]. The emergence of high-throughput technologies has revolutionized our ability to map molecular interactions within cells, giving rise to the concept of the interactome—the comprehensive set of molecular interactions within a specific cell, tissue, or organism [2,3,4]. The interactome encompasses protein–protein interaction (PPIs), protein-DNA interactions, protein-RNA interactions, and metabolic networks, forming a complex web of relationships that regulate cellular and organ function [5,6,7,8].

Traditional methods for constructing interactomes have focused on identifying stable and reproducible interactions under controlled conditions. These approaches generally seek to minimize variability and noise, regarding them as experimental artifacts that obscure the genuine biological signal [9,10,11]. Most interactome models are static and binary, which stems from their design and data limitations rather than an intention to eliminate variability. High-throughput screening methods have generated extensive datasets of molecular interactions, leading to the establishment of comprehensive databases, such as BioGRID, STRING, IntAct, and MINT [12,13,14]. The reductionist approach often overlooks the dynamic and variable nature of biological systems, in which cellular states constantly fluctuate in response to both internal and external stimuli [15,16]. Increasing evidence suggests that biological systems are inherently noisy and variable, with this variability playing essential functional roles rather than merely representing experimental error [15,16].

The Constrained Disorder Principle (CDP) challenges the conventional paradigm by proposing that controlled variability and biological noise are not obstacles to overcome, but rather fundamental features of living systems that should be incorporated into our models [17].

The CDP suggests that biological systems operate within a framework of constrained randomness, where variability serves essential functional roles while remaining bounded by physiological limits [18,19,20,21,22]. It has significant implications for interactome research, indicating that accurate models must account for the random nature of molecular interactions, the temporal dynamics of cellular states, and the inherent variability across individuals, cell types, and environmental conditions. By incorporating these factors, the CDP provides a pathway to more accurate, biologically relevant interactome models that better predict cellular behavior and inform therapeutic strategies.

This paper examines how the CDP can potentially enhance the accuracy of interactome models by accounting for the dynamic and variable nature of biological systems while upholding functional constraints. It describes several case studies in which incorporating controlled variability into interactome models may significantly enhance their predictive power and biological relevance.

## 2. The Traditional Interactome: Achievements and Limitations

### 2.1. Historical Development and Current Methodologies

Initial studies in interactomics were primarily small-scale and hypothesis-driven, focusing on specific protein complexes or pathways. The advent of high-throughput screening methods has transformed the field, enabling the systematic mapping of interaction networks on a genome-wide scale [23]. Yeast two-hybrid (Y2H) screening was one of the first techniques to enable large-scale interaction mapping. This method identifies protein–protein interaction by reconstituting a functional transcription factor through the interaction of fusion proteins in yeast cells [24]. However, Y2H has difficulty detecting transient interactions, is prone to false positives due to artificial protein expression levels, and cannot identify interactions that require post-translational modifications. Affinity purification combined with mass spectrometry (AP-MS) has become a complementary technique, enabling the identification of protein complexes under more physiologically relevant conditions [25]. This technique entails expressing tagged bait proteins, purifying related protein complexes, and identifying their components through mass spectrometry. AP-MS can capture indirect interactions and provide insights into the stoichiometry of protein complexes, though it may miss transient or weak interactions [26,27,28].

Computational prediction methods have played vital roles in constructing interactomes. These approaches utilize evolutionary conservation, protein structural data, gene expression trends, and functional annotations to predict potential interactions [29,30,31]. While these methods can generate interaction predictions and help validate experimental findings, they rely heavily on the quality and completeness of the available data. Large-scale initiatives, such as the Human Protein Reference Database (HPRD), the Biological General Repository for Interaction Datasets (BioGRID), and the Search Tool for the Retrieval of Interacting Genes/Proteins (STRING), have developed extensive interaction databases by combining information from various experimental and computational sources [32,33,34,35].

### 2.2. Major Achievements

Although traditional interactome methods have their limitations, they have achieved significant success in various areas. Network architecture studies have revealed key principles of biological network organization, including scale-free topology, modular structures, and the frequent presence of hub proteins that interact with numerous partners [36]. The finding that biological networks exhibit small-world properties, characterized by short path lengths between any two nodes, has shed light on how information can spread quickly through cellular systems [37,38,39].

Interactome data have enabled the identification of disease genes through guilt-by-association methods, in which genes linked to known disease genes are considered potential candidates for disease [40]. Interactome data have been crucial in reconstructing biological pathways and in understanding how cellular processes are organized into functional modules. This has improved our understanding of fundamental processes, such as signal transduction, metabolic regulation, and gene expression control [24].

Network-based approaches have identified new drug targets and clarified the mechanisms of action. The concept of network pharmacology has emerged, recognizing that drugs often act on multiple targets within interaction networks rather than on isolated proteins [41,42,43].

### 2.3. Fundamental Limitations

Despite these accomplishments, traditional interactome approaches face several limitations that undermine their biological accuracy and practical utility.

Static Representation: Most interactome models depict interactions as binary, static relationships—either they exist, or they do not. This binary representation fails to capture the continuous nature of interaction strengths, the temporal dynamics of binding events, and the context-dependent nature of molecular associations. In reality, protein interactions exist along a spectrum of binding affinities and can be influenced by various factors, including post-translational modifications, allosteric regulation, and competitive binding [44,45,46].

Context Independence: Traditional interactomes often combine data from various experimental conditions, cell types, developmental stages, and even different organisms, resulting in average networks that may not accurately reflect any specific biological context [47]. This averaging effect can obscure significant context-specific interactions and establish misleading connections between proteins that do not actually coexist in the same cellular compartment or temporal window [48,49,50].

Noise Exclusion: Standard methodologies employ strict statistical cutoffs and reproducibility requirements, often leading to the exclusion of potentially significant low-frequency or variable interactions. This approach assumes that reproducible interactions are more biologically relevant than variable ones. However, this assumption may not apply to all biological processes [51]. Such approaches may overlook rare but functionally important interactions involved in stress responses or developmental processes. In many cases, this may be due to limited data, which imposes a specific design on the algorithms [51,52,53].

Temporal Blindness: Many interactome studies provide snapshots of molecular interactions at specific time points, overlooking the temporal dynamics essential for understanding cellular processes, such as signaling cascades, cell cycle progression, and developmental programs [54]. The dynamic nature of protein complexes, where their components can associate and dissociate over timescales ranging from milliseconds to hours, is often overlooked in static network representations [1,38,55].

Individual Variation Neglect: Traditional approaches often combine data from multiple individuals or experimental replicates, thereby obscuring significant inter-individual variability. This averaging may obscure crucial genetic variants that influence protein interactions, epigenetic modifications that affect binding patterns, and environmental factors that alter interaction networks [56,57,58].

Incomplete Coverage: Current interactome datasets have significant coverage gaps, with estimates indicating that only a small fraction of all possible protein–protein interaction have been experimentally detected [59]. This incomplete coverage is particularly noticeable for specific protein classes, including membrane proteins, transcription factors, and lowly expressed proteins [60,61].

Methodological Biases: Different experimental techniques come with inherent biases that can distort interaction datasets. For instance, yeast two-hybrid (Y2H) screens are likely to favor the detection of strong, direct interactions, potentially overlooking weaker or indirect associations. In contrast, affinity purification-mass spectrometry (AP-MS) methods may be more adept at identifying stable protein complexes, but they may miss transient interactions [62,63,64].

### 2.4. Consequences of These Limitations

These limitations significantly impact the biological utility and translational potential of interactome data.

**Reduced Predictive Power:** Static, averaged networks often struggle to predict cellular behavior under specific conditions or in response to disturbances. This limitation reduces their effectiveness in understanding disease mechanisms, predicting drug responses, and developing therapeutic interventions [65].

**Limited Clinical Relevance:** Interactomes that ignore individual variability may not accurately reflect the molecular networks operating in specific patients or disease states. This diminishes their value for personalized medicine approaches and can lead to therapeutic strategies that work well on average but fail for some individual patients [2,66,67].

**Oversimplified Disease Models:** When based on oversimplified network models, disease mechanisms may be misinterpreted, as these models fail to account for the dynamic and variable nature of pathological processes. This can result in an incomplete understanding of disease progression and ineffective therapeutic strategies [68,69,70].

**Missed Therapeutic Opportunities:** Important drug targets or combination therapies may be overlooked if they involve variable or context-specific interactions that traditional approaches do not capture [71,72].

## 3. The Constrained Disorder Principle: Theoretical Foundation

### 3.1. Conceptual Framework

The Constrained Disorder Principle (CDP) represents a significant shift in our understanding of biological systems. Instead of seeing variability and noise as issues to be eliminated, the CDP acknowledges these elements as essential characteristics of living systems that play crucial functional roles [17]. This principle challenges the traditional reductionist view, which seeks to identify laws and universal mechanisms underlying complex phenomena. It proposes that biological systems operate based on principles that explicitly incorporate randomness and variability within defined constraints [17,73,74,75,76,77,78]. It is based on the concept of the critical zone of variability, as previously described [79].

The CDP is based on several key concepts that differentiate it from traditional biological modelling approaches.

**Functional Variability:** Biological systems exhibit variability across scales, from molecular interactions to physiological processes. Per the CDP, this variability is purposeful, serving specific functions such as adaptation to environmental changes, exploration of phenotypic diversity, and maintenance of system robustness [18,19,20,21,80,81,82,83,84,85,86,87,88,89,90]. In contrast to engineering systems, which are typically designed to minimize variability, biological systems have evolved to exploit variability as a functional advantage [14,53].

**Constrained Randomness:** According to the CDP, biological systems exhibit significant variability, but within specific boundaries that ensure their viability and functionality. These boundaries can be physical (e.g., thermodynamic limits), chemical (e.g., reaction stoichiometry), or regulatory (e.g., feedback control mechanisms). The interaction between randomness and these constraints fosters a dynamic balance that allows for both stability and flexibility [91,92,93].

**Dynamic Equilibrium:** Per the CDP, biological systems operate in a state of dynamic equilibrium, characterized by constant fluctuations around dynamic attractors. These fluctuations are not merely deviations from an ideal state; instead, they are essential components of the system’s function. They enable adaptations to environmental changes and help maintain proper functionality [94,95,96].

**Hierarchical Organization:** The CDP acknowledges that biological variability and constraints operate simultaneously across multiple scales. Noise at the molecular level can influence cellular behavior, while constraints at higher levels can impact processes at lower levels [24,94,97].

**Context Dependence:** The CDP accounts for patterns of variability and constraint that depend on context, meaning they differ across cell type, developmental stage, environmental conditions, and individual genetic backgrounds. This context dependence suggests that no single “correct” network structure exists; instead, a range of possible configurations varies with biological context [98].

**Evolutionary Optimization:** The CDP posits that evolution has not only refined the average behavior of biological systems but also their patterns of variability. This implies that the noise and fluctuations we observe are not merely accepted; they have been actively selected for their functional advantages [22,89,98].

**The information that is stored within systems**: According to the CDP, the inherent variability of systems is a crucial aspect of information in complex systems. Therefore, biological noise should not be viewed as an error; instead, it carries information and is fundamental for the proper functioning of these systems. It is essential to consider this variability when analyzing such systems [96].

**Systems structure:** According to the CDP, noise is present at all levels of the system, from the atomic to the organ levels. For example, heart rate variability, blood pressure variability, and glycemic variability are essential for proper physiological functioning. This principle suggests that low levels of variability, out-of-range variability, or a lack of dynamism in system variability are associated with malfunctions and disease conditions [97,99,100,101,102,103,104,105,106,107,108,109,110,111,112,113,114,115,116,117,118,119,120,121,122,123,124,125,126].

### 3.2. Mathematical Formulation

The CDP can be mathematically represented using concepts from statistical mechanics and information theory. Let us consider a biological system whose state is described by a vector (x) in a high-dimensional state space. In the CDP framework, the accessible state space is defined as follows:

S = {x | C(x) ≤ θ}

where C(x) denotes a constraint function that encompasses the essential requirements for system viability and functionality, and θ signifies the constraint threshold. This constraint function may integrate various types of constraints, such as:

- Energy constraints: Ensuring the system stays within thermodynamically feasible states.
- Stoichiometric constraints: Maintaining proper ratios of molecular components.
- Regulatory constraints: Meeting feedback control requirements.
- Spatial constraints: Respecting cellular compartmentalization.

Within this limited space, the system displays a form of controlled randomness, which is defined by a specific probability distribution:

P(x) = f(x) × g(C(x))/Z

where

- P(x) is the probability of observing state x
- f(x) represents the intrinsic variability function, capturing the natural tendencies of the system
- g(C(x)) represents the constraint weighting function, which modulates probability based on how well constraints are satisfied
- Z is the normalization constant ensuring that probabilities sum to unity

This formulation enables the incorporation of various sources of variability while adhering to essential system constraints. The balance between exploration (driven by f(x)) and constraint satisfaction (modulated by g(C(x))) determines the system’s behavior.

For molecular interaction networks, this framework can be expanded to depict interaction probabilities as functions of both intrinsic molecular properties and contextual constraints:

P(i,j|context) = f_ij(molecular properties) × g_ij(context constraints)/Z_context

where P(i,j|context) represents the probability of interaction between molecules i and j in a given context.

This formulation accounts for the system’s variability dynamics while preserving the overall integrity of complex systems.

### 3.3. Information-Theoretic Perspective

From an information–theoretic perspective, the CDP can be seen as optimizing the balance between information processing capacity and the need to satisfy constraints. Biological systems must process information about their environment while also maintaining essential functions. If there is too much order (low variability), it limits information-processing capacity; conversely, too much disorder (high variability) can compromise functional reliability [22,91,92,93,95,96].

The CDP indicates that biological systems, while maintaining their integrity, operate near a critical point that maximizes information-processing capacity while satisfying functional constraints. This critical behavior has been observed in neural networks and may represent a universal principle of biological organization [13,84].

### 3.4. Biological Implications

The CDP has several significant implications for understanding biological systems and addressing system malfunctions.

**Adaptive Capacity:** Variability allows biological systems to explore various functional states and adapt to changing conditions without requiring genetic modifications. This phenotypic plasticity is crucial in rapidly changing environments, where genetic adaptation may be too slow [22,89,98].

**Robustness:** Controlled variability can enhance system resilience by preventing suboptimal states and providing multiple pathways to achieve essential functions [97,99,100,101,102,103,104,105,106,107,108,109,110,111,112,113,114,115,116,117,118,119,120,121,122,123,124,125,126].

**Information Processing:** Variability can provide valuable information, enabling cells to sense and respond to subtle changes in their environment that might otherwise go unnoticed in purely deterministic systems [13,38,51,52,53,55]. One example of how variability can enhance information processing is stochastic resonance, in which noise improves signal detection.

**Evolutionary Evolvability:** Systems that integrate controlled variability may be more evolvable, as they can explore phenotypic space more effectively and respond to selective pressures more easily [13,49,56].

**Overcoming System Malfunctions:** The CDP-based second-generation artificial intelligence system has demonstrated its ability to overcome drug tolerance and enhance clinical outcomes by incorporating regulated variability into treatment regimens and diagnostic procedures. This supports the CDP concept of utilizing variability to improve effectiveness [73,76,78,108,123,126,127,128,129,130,131,132,133,134].

**Figure 1:**
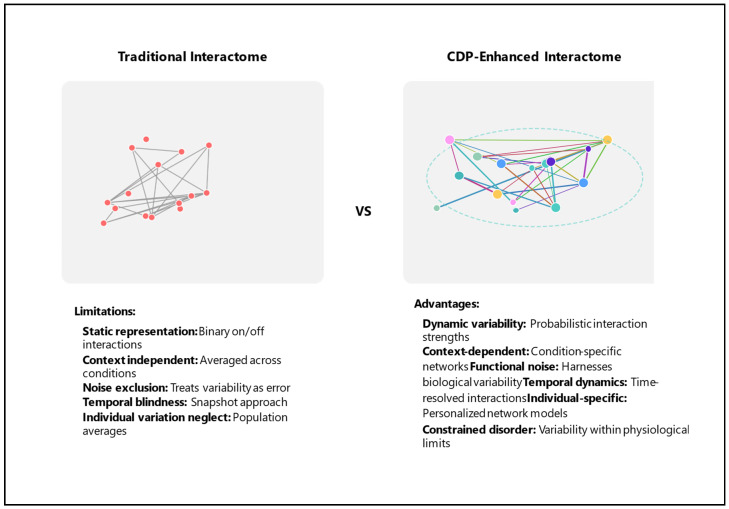
A schematic description of the differences between the traditional interactome and the CDP-enhanced interactome.

**Figure 2:**
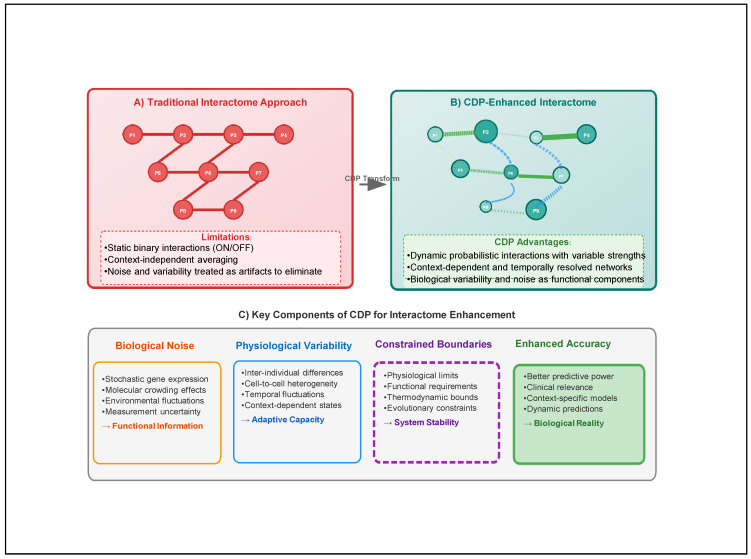
A schematic overview of key components of the CDP for interactome enhancement.

Figure 1 highlights some key differences between the standard interactome and the CDP-enhanced interactome. Figure 2 presents a schematic overview of key components of the CDP for interactome enhancement. In both instances, the system’s overall integrity is preserved.

## 4. Applying the Constrained Disorder Principle to Interactome Analysis

### 4.1. Reconceptualizing Molecular Interactions

The application of the CDP to interactome analysis necessitates a fundamental rethinking of molecular interactions. Instead of viewing these interactions as binary events, the CDP framework understands them as probabilistic processes defined by multiple continuous parameters, while preserving system integrity.

**Interaction Probability and Strength:** The likelihood of two molecules interacting depends on specific conditions, such as their concentrations, cellular locations, competing interactions, and the presence of cofactors or inhibitors. This probability is not constant; it changes dynamically in response to cellular context, environmental conditions, and the presence of competing interactions, cofactors, or inhibitors [135]. This probability is not fixed; it varies dynamically in response to cellular context and environmental conditions [31,135,136].

**Temporal Dynamics:** The interactions between molecules change over time, affecting both association and dissociation rates and the duration of their functional coupling. Some interactions may be constant, while others are temporary and occur only under specific conditions or during cellular processes [137,138].

**Context Sensitivity:** The interaction parameters depend on cellular context, which includes metabolic state, stress conditions, developmental stage, and tissue type. The same pair of proteins may interact differently across various contexts, exhibiting varying probabilities, strengths, and functional consequences [139,140].

**Functional Multiplicity:** The same molecular interaction can have different functional consequences based on context. For instance, a protein–protein interaction may be activated in one scenario but inhibited in another, influenced by the presence of other factors or post-translational modifications [141,142].

The CDP accounts for the reconceptualization shift from viewing interactions as fixed edges in a static graph to understanding them as dynamic, context-dependent processes within a continuous space of possibilities [143]. These concepts, which are valid for molecular interactions, are also applicable to cellular and organ functionality.

### 4.2. Incorporating Physiological Variability

The CDP framework explicitly incorporates physiological variability into interactome models while ensuring the integrity of the biological system.

**Inter-individual Variation:** The CDP accounts for the fact that interaction networks can vary significantly among individuals due to factors such as genetic polymorphisms, epigenetic modifications, and environmental exposures [144]. Single-nucleotide polymorphisms (SNPs) can affect the structure, stability, or expression levels of proteins, leading to individual differences in how these proteins interact. Additionally, epigenetic modifications can influence gene expression, thereby altering the abundance of interacting proteins.

**Cell-to-Cell Heterogeneity:** Even genetically identical cells within the same tissue can display significant differences in their molecular interaction patterns. This variability, which is accounted for by the CDP, can arise from factors such as random gene expression, differences in the cell cycle, and variations in the microenvironment. Single-cell studies have demonstrated considerable heterogeneity in gene expression patterns, even within populations considered homogeneous [145].

**Temporal Fluctuations:** The CDP integrates time-dependent changes in interaction networks influenced by circadian rhythms, cell cycle progression, environmental stimuli, and developmental programs. Proteins may interact at specific times during the cell cycle or in response to signals, and these temporal patterns are crucial for proper cellular function.

**Condition-Specific Networks:** The development of CDP-based context-specific interactome models aims to capture the unique interaction patterns observed across various physiological and pathological conditions. For instance, stress-response networks may exhibit interactions that are absent under normal conditions, while metabolic networks can reorganize in response to changes in nutrient availability.

**Spatial Heterogeneity:** The CDP acknowledges that cellular compartmentalization limits molecular interactions. Proteins located in different subcellular compartments cannot interact directly, and the movement of proteins between these compartments can dynamically influence interaction patterns [146].

### 4.3. The CDP Accounts for Embracing Biological Noise

Instead of viewing biological noise as an experimental artifact to be minimized, the CDP framework acknowledges it as a key characteristic of biological systems that should be integrated into interactome models.

**Stochastic Gene Expression:** Gene expression is inherently noisy, resulting in variability in protein levels and interaction probabilities between cells. This variability is due to the discrete nature of molecular events and the low abundance of many regulatory molecules [147,148,149].

**Molecular Crowding Effects:** Incorporating molecular crowding and competitive binding introduces stochastic elements into molecular interactions, thereby increasing their complexity. In the crowded environment of the cell, interactions can be influenced by random encounters and competitive binding events [31,150].

**Environmental Fluctuations:** It is essential to investigate how environmental noise and fluctuations impact molecular interaction patterns. Variations in temperature, pH, and metabolite concentrations significantly influence the probabilities and strengths of these interactions [96,151,152].

**Functional Roles of Noise:** Recognizing that biological noise can fulfill functional roles, such as facilitating cell fate decisions, enhancing signal detection through stochastic resonance, or contributing to phenotypic diversity beneficial for natural selection [153,154].

**Measurement Noise vs. Biological Noise:** It is essential to distinguish between true biological variability and measurement noise, recognizing that both contribute to the observed patterns in experimental data. Advanced statistical methods can help separate these sources of variability [155].

### 4.4. Dynamic Network Models

The CDP facilitates the creation of dynamic interactome models that reflect the temporal evolution of molecular interaction networks.

**State-Dependent Networks:** According to the CDP, networks can change their configuration based on cellular states, activating different interaction patterns depending on the conditions [156].

**Transition Dynamics:** According to CDP-based models, networks undergo changes in their configurations in response to external stimuli. These changes can involve forming new interactions, disrupting existing ones, or altering the strengths of interactions.

**Equilibrium Fluctuations:** According to the CDP, the actual equilibrium always involves a certain degree of variability that is essential for proper functionality [95]. Even in steady-state conditions, networks exhibit continuous dynamics as interactions form and dissolve [157].

**Memory Effects:** The CDP perceives memory as integrated within the dynamic boundaries of variability, serving as a mechanism for adapting to both internal and external disruptions. Cells can demonstrate molecular memory through mechanisms such as persistent post-translational modifications or stable protein complexes [158].

**Multi-Scale Dynamics:** According to the CDP, network dynamics occur on various timescales simultaneously, ranging from rapid molecular association events (milliseconds) to slower processes, such as protein synthesis and degradation (minutes to hours).

## 5. Methodological Advances for CDP-Based Interactomics

### 5.1. Experimental Approaches

Implementing the CDP in interactome research necessitates new experimental methods that can capture variability and dynamics.

**Single-Cell Technologies:** Single-cell RNA sequencing (scRNA-seq), single-cell proteomics, and single-cell imaging techniques provide the necessary resolution to observe molecular interaction variability at the cell-by-cell level. These technologies have revealed significant heterogeneity within cellular populations and have enhanced our understanding of the functional roles of this variability [159]. Single-cell multiomics enable the simultaneous measurement of multiple molecular layers—transcriptome, epigenome, and proteome—within individual cells, providing comprehensive insights into cellular state variability. Techniques for mapping single-cell interactions, such as proximity ligation assays tailored for individual cells, can facilitate direct measurements of variability in these interactions [160,161,162].

**Time-Resolved Measurements:** Temporal profiling techniques capture the dynamics of molecular interactions across multiple time scales, from seconds to days. Live-cell imaging with fluorescently tagged proteins enables real-time observation of protein–protein interaction and their dynamics. Time-resolved mass spectrometry methods can monitor changes in the composition of protein complexes over time, providing insights into their assembly and disassembly dynamics. Additionally, pulse-chase experiments using stable isotope labeling can provide valuable insights into protein turnover rates and their impact on interaction networks [163,164].

**Perturbation Studies:** Systematic perturbation experiments explore how interaction networks respond to different stimuli, revealing their dynamic properties and regulatory mechanisms. These perturbations can be chemical (such as drug treatments), genetic (including knockouts and knockdowns), or environmental (such as stress conditions and changes in nutrient levels) [165]. CRISPR-based screening techniques enable systematic manipulation of genes and regulatory elements to assess their impacts on network structure and dynamics. Optogenetic tools allow precise control of protein activity, enabling the accurate manipulation of interaction networks [165,166].

**Multi-Modal Integration:** Integrating various molecular measurements builds comprehensive views of interaction networks and their variability. The combination of genomics, transcriptomics, proteomics, and metabolomics data provides holistic insights into cellular states and their fluctuations. Cross-linking mass spectrometry (XL-MS) provides structural insights into protein complexes and their dynamics, complementing conventional interaction detection methods. Cryo-electron microscopy is increasingly used to study protein complex structures and conformational dynamics [167,168,169].

### 5.2. Computational Methods

The CDP requires new computational methods for analyzing and modeling data related to variable interactomes.

**Probabilistic Network Models:** Bayesian networks and other probabilistic models can represent uncertainty and variability in molecular interactions. These models incorporate prior knowledge about biological systems while allowing for uncertainty and learning from data. Gaussian graphical models and their extensions effectively represent both continuous interaction strengths and their variability. Hidden Markov models can accurately capture the temporal dynamics in interaction patterns. Factor graphs provide a flexible framework for modeling complex dependencies among molecular components [170,171].

**Machine Learning Approaches:** Deep learning and machine learning methods are effective at identifying patterns in high-dimensional, noisy interactome data. Neural networks can learn complex nonlinear relationships between cellular contexts and interaction patterns. Graph neural networks are well-suited for analyzing network data, as they can incorporate both node features (e.g., protein properties) and the network structure. Autoencoders can learn to create low-dimensional representations of high-dimensional interaction data, helping to uncover underlying patterns and structures [172,173,174].

**Dynamic Network Algorithms:** Computational methods are used to infer and analyze time-varying networks from temporal data. Dynamic network models can monitor how network structure changes over time and identify key transition points or regulatory events. Network alignment algorithms compare network structures under different conditions or at various time points, highlighting conserved and variable interaction patterns [175]. Additionally, community detection algorithms tailored for temporal networks can identify functional modules and track their evolution over time [176,177].

**Multi-Scale Modeling:** Integrating molecular-level variability with higher-level biological processes can be achieved through multi-scale modeling. These models link molecular interaction dynamics to cellular behavior and physiological outcomes [178,179]. Agent-based models simulate the behavior of individual molecular components and their interactions, allowing for the exploration of how molecular-level variability impacts system-level properties. Additionally, flux balance analysis and its extensions can incorporate variability in interactions into metabolic network models [179,180].

### 5.3. Data Integration Strategies

The CDP framework requires novel methods to integrate diverse types of interactome data while preserving the integrity of complex systems.

**Heterogeneous Data Fusion:** Methods for integrating data from various experimental platforms and conditions while maintaining information about variability require careful attention to platform-specific biases and sources of variability [181]. Multi-view learning approaches can synthesize data from different experimental methods, each offering a unique perspective on the interaction network. Tensor factorization methods can analyze multiple data types and conditions simultaneously, identifying both shared patterns and variations specific to certain conditions [182].

**Uncertainty Quantification:** Techniques for propagating and quantifying uncertainty through complex data integration pipelines are essential for making reliable inferences from noisy and incomplete data. Bootstrap and jackknife resampling methods can be used to provide confidence intervals for network parameters. Bayesian approaches are practical for quantifying uncertainty and can integrate prior knowledge about biological systems. Additionally, ensemble methods enhance prediction robustness by combining multiple models [183,184].

**Context-Aware Integration:** Methods that maintain the context-dependence of interactions during data integration are preferred over generating a single average network. These approaches preserve information about how interactions change across different contexts [185,186]. Multi-layer network models can represent various types of interactions and their interdependencies. Context-specific network reconstruction algorithms can construct separate networks for different conditions while also identifying shared components [186].

**Personalized Network Construction:** Methods for constructing personalized interaction networks that consider both genetic and environmental factors are essential for personalized medicine applications. Genome-wide association studies (GWAS) can reveal genetic variants that influence interaction patterns [187,188]. Additionally, quantitative trait locus (QTL) mapping can link genetic variation to molecular phenotypes, including protein interactions. Machine learning techniques can also be employed to predict individual-specific network characteristics from genetic and environmental data [189,190].

## 6. The CDP Accounts for the Importance of Variability in Biological Systems

### 6.1. Molecular Level Evidence

There is significant evidence that highlights the importance of molecular variability within complex systems. This variability does not contradict the integrity of complex systems and, according to the CDP, is considered essential for proper functionality.

**Gene Expression Noise:** Noise in gene expression is not only tolerated but also actively regulated, playing a significant functional role. Gene expression noise comprises both intrinsic noise, arising from the stochastic nature of transcription and translation, and extrinsic noise, stemming from variations in cellular machinery across cells. This noise can influence cell-fate decisions, allowing cells to utilize stochastic fluctuations to explore various developmental pathways [15,147]. Noise levels vary among different genes and are evolutionarily optimized for specific functions. Genes involved in stress responses tend to exhibit higher noise levels, allowing for a quicker exploration of adaptive responses. In contrast, housekeeping genes typically exhibit lower noise levels, which help maintain stable cellular functions [191].

**Protein Folding Dynamics:** Proteins exist as dynamic ensembles of conformations rather than as static structures. This conformational variability is often crucial for their function. Proteins continuously sample various conformational states. Binding events frequently involve mechanisms of conformational selection or induced fit [192]. Allosteric regulation depends on these conformational dynamics, as binding at one site can influence the conformation and activity at a distant site. This type of regulation would not be possible without protein flexibility and conformational variability [193,194].

**Enzyme Kinetics:** Single-molecule studies have shown that individual enzyme molecules exhibit significant variability in their kinetic properties, which can enhance catalytic efficiency under specific conditions. Static disorder occurs when different enzyme molecules have varying reaction rates, leading to a broader distribution of activity that can be beneficial in fluctuating environments [195,196]. On the other hand, dynamic disorder involves individual enzymes switching between different activity states over time. This phenomenon has been observed in many systems and may play a role in enzymatic regulation and adaptation. The switching behavior of enzymes can be influenced by environmental conditions or regulatory molecules [195,196].

**DNA-Protein Interactions:** The binding of transcription factors to DNA is highly variable, with binding events stochastic and context-dependent. Transcription factors continuously associate with and dissociate from DNA, with binding durations ranging from seconds to minutes [197]. This dynamic binding behavior is crucial for gene regulation, enabling rapid responses to environmental changes while maintaining regulatory specificity. The unpredictable nature of transcription factor binding adds to gene expression variability and may facilitate exploration of different regulatory states [198,199].

### 6.2. Cellular Level Evidence

At the cellular level, variability has been shown to play a crucial role.

**Bet-Hedging Strategies:** Cellular populations often display phenotypic variability as a bet-hedging strategy, ensuring that some individuals can survive environmental disturbances [200,201]. This strategy is prominent in microbial systems, where genetically identical cells exhibit significantly different phenotypes [201]. In yeast, cells exhibit variable expression of stress-response genes even under normal conditions, resulting in subpopulations that are pre-adapted to different types of stress. This variability enhances the population’s survival amid unpredictable environmental changes [202,203].

**Signal Processing:** Cellular noise can enhance signal detection and processing, enabling cells to respond to weak stimuli that might be overlooked in strictly deterministic systems. This phenomenon, known as stochastic resonance, has been observed in various biological systems [204,205]. For instance, in bacterial chemotaxis, noise within the signaling pathway can improve the detection of weak chemical gradients. In mammalian cells, signaling pathway noise enhances the sensitivity of cell cycle checkpoints and apoptotic decision-making [206].

**Differentiation and Development:** Cell-to-cell variability in gene expression patterns plays a crucial role in cellular differentiation and developmental processes [207]. Stochastic fluctuations can push cells beyond critical thresholds, leading to a commitment to specific cell fates. In hematopoiesis, fluctuations in transcription factor levels influence the differentiation of stem cells into various blood cell lineages. Similar mechanisms are present in many other developmental systems as well [208,209].

**Circadian Rhythms:** Biological clocks display a controlled variability that serves functional roles [210]. While circadian rhythms must maintain a consistent timing, a degree of variability in their period lengths permits adaptation to seasonal changes and personal lifestyle differences.

### 6.3. Systems Level Evidence

Variability has functional significance at the systems level and contributes to systems integrity. It can be measured by analyzing heart rate variability, blood pressure variability, neuronal variability, glucose variability, gait variability, and others [211,212,213].

**Network Robustness:** Variable network configurations can improve resilience to disruptions and failures [214]. Scale-free networks, commonly observed in biological systems, exhibit strong resistance to random node failures but are vulnerable to targeted attacks on their highly connected nodes. By introducing variability, these networks can enhance their robustness, providing alternative pathways and redundant connections [215]. Research on metabolic networks suggests that organisms exhibiting greater variability in enzyme expression tend to be more resilient to environmental changes. This implies that such variability acts as a biological safeguard against unforeseen challenges [216,217,218].

**Adaptive Responses:** Variability allows populations of cells or organisms to adapt more effectively to changing environments than homogeneous populations [219]. In bacterial populations, phenotypic variability facilitates rapid adaptation to antibiotic treatments, as resistant subpopulations can emerge from this diverse population [220]. Cancer cell populations exhibit significant variability, which contributes to the development of therapeutic resistance. Tumor heterogeneity, influenced by both genetic and epigenetic factors, enables the emergence of drug-resistant clones [221].

**Disease Mechanisms:** Many diseases involve disruptions in the standard patterns of biological variability, suggesting that proper regulation of this variability is crucial for maintaining health. As we age, our cells lose their ability to maintain appropriate levels of variability, resulting in increased noise in some systems and reduced adaptability in others [96]. Neurodegenerative diseases often disrupt normal noise regulation in neural networks. In Alzheimer’s disease, abnormal protein aggregation disrupts synaptic plasticity, leading to cognitive decline [222].

**Immune System Function:** The immune system is an example of a biological system that utilizes variability for its function. The creation of diverse antibody and T-cell receptor repertoires through V(D)J recombination generates significant variability, enabling the recognition of virtually any pathogen [223,224]. In addition to developing an initial repertoire of immune responses, the immune system employs controlled variability through processes such as somatic hypermutation and affinity maturation to enhance its responses [126].

According to the CDP, it demonstrates how biological systems can use variability not only for resilience but also for active optimization and learning. Variability is essential for maintaining the integrity and functionality of complex systems. Health issues and system malfunctions arise from insufficient variability.

## 7. Case Studies Demonstrating How CDP-Enhanced Interactome Analysis Could Potentially Address System Malfunctions

### 7.1. Cancer Network Dynamics

Cancer serves as an excellent case study for applying the CDP to interactome analysis, as it involves dynamic changes in molecular interaction networks driven by genetic instability and selective pressure [225].

**Tumor Heterogeneity:** The CDP framework addresses the significant diversity within tumors, where various cells exhibit distinct molecular interaction patterns. Traditional methods that average tumor samples may overlook crucial subclonal networks that drive disease progression or resistance to therapy. Single-cell studies of tumors have uncovered significant variability in gene expression patterns, metabolic states, and signaling pathway activities within individual tumors [226,227]. This variability is not merely noise; it represents distinct cellular adaptations to the tumor microenvironment and different evolutionary paths taken by the tumor cells [228].

**Resistance Mechanisms:** By incorporating variability, CDP-based models can improve the prediction and understanding of drug resistance, which often involves rare cellular states that traditional approaches may overlook. Drug resistance typically arises from pre-existing resistant subpopulations or from random fluctuations that allow specific cells to survive treatment. For instance, in melanoma treated with BRAF inhibitors, resistance frequently develops through the activation of alternative signaling pathways [229]. CDP-based models could identify variable interaction patterns that facilitate these alternative pathways and predict combination therapies targeting multiple network configurations.

**Therapeutic Implications:** CDP-enhanced interactome models can aid in developing therapeutic strategies that account for tumor variability and dynamics. Instead of focusing on single pathways, these approaches may aim to limit the range of viable network configurations or capitalize on the costs associated with maintaining high variability. Combination therapies designed using CDPs could target both the average network configuration and the variable components that contribute to adaptation. This strategy may help prevent or delay the emergence of resistance by limiting the evolutionary options available to cancer cells.

**Metastatic Progression:** The CDP framework has the potential to offer insights into metastatic progression by modeling how interaction networks must adapt to facilitate invasion, survive in circulation, and establish colonies in distant sites. Each stage of the metastatic cascade may require distinct network configurations, and successful metastases must maintain enough variability to adjust to new environments [230,231].

### 7.2. Neurodegenerative Diseases

Neurodegenerative diseases encompass complex, progressive changes in molecular interaction networks that unfold over years or decades.

**Disease Progression:** The CDP can model the gradual changes in interaction networks during disease progression while accounting for variability in disease trajectories among individuals. Different patients may follow distinct pathways of network degradation, contributing to the observed differences in disease symptoms and progression rates. In Alzheimer’s disease, the buildup of amyloid plaques and neurofibrillary tangles disrupts normal synaptic networks in both spatial and temporal ways. CDP-based models can be used to track how these disruptions spread through brain networks and to identify key network components whose failure may lead to cognitive symptoms [77,126].

**Protein Aggregation:** The stochastic nature of protein aggregation processes in diseases such as Alzheimer’s and Parkinson’s can be better understood through CDP-based models [232,233]. Protein aggregation involves inherently random nucleation events, followed by growth phases that can exhibit complex dynamics. The CDP framework can model how aggregation events interact with normal cellular networks, helping to identify the mechanisms through which aggregates disrupt everyday protein interactions and cellular functions. This understanding could lead to therapeutic strategies that target the aggregation process at critical points within these networks [78].

**Therapeutic implications:** By recognizing individual differences, CDP models could help determine the optimal therapeutic windows for various patients. The same treatment may work effectively at different stages of a disease for other individuals, depending on their specific network configurations and patterns of disease progression. CDP-based models could identify biomarkers of network states that indicate when therapies would be the most effective. Second-generation AI systems using CDP improved treatments for neurodegenerative diseases, including multiple sclerosis, Parkinson’s disease, aging processes, and chronic pain by incorporating regulated variability into therapeutic regimens [78,131,234].

**Resilience and Reserve:** The concept of cognitive reserve suggests that some individuals can maintain their mental function even in the presence of significant brain pathology [235]. CDP-based models can help explain this phenomenon by identifying network configurations that are resilient to pathological changes. Additionally, these models may slow the aging process by incorporating variability into activities.

### 7.3. Immune System Function

The immune system exhibits significant variability and dynamics, making it a prime target for CDP [126].

The generation of diverse antibody and T-cell receptor repertoires involves controlled randomness that is essential for immune function. V(D)J recombination generates immense diversity by randomly combining gene segments, while somatic hypermutation introduces further variability during immune responses [236]. CDP-based models could optimize the balance between diversity generation and functional constraint, identifying the mechanisms that ensure sufficient diversity while avoiding autoimmunity. These models could also predict how repertoire diversity changes with age or disease [89,90].

The formation and maintenance of immunological memory involve dynamic changes in interaction networks that must strike a balance between stability and flexibility [237,238]. Stability is essential for retaining memory, while flexibility enables an effective response to new threats. Memory B and T cells exist in different activation states and can quickly reorganize their interaction networks upon re-encountering an antigen [239]. According to the CDP, memory is embedded within the dynamic boundaries that determine the variability range of a system [98].

Per the CDP, autoimmune diseases may arise from disruptions in the normal variability of immune networks. A loss of tolerance mechanisms could result in either excessive variability or insufficient variability in immune responses. Conversely, overactive tolerance mechanisms could diminish the variability necessary for effective pathogen recognition.

CDP models can identify the network constraints that maintain immune tolerance and predict how genetic or environmental factors might disrupt these constraints. This may lead to new therapeutic approaches that restore appropriate levels of immune variability. Insights into immune network variability could guide vaccination strategies. Different individuals may need tailored vaccine formulations or schedules based on their unique immune network configurations. CDP-based methods could facilitate personalized vaccination strategies that consider individual differences in immune system organization [85].

Future research could further investigate these concepts to enhance the implementation of CDP-based interactomes, thereby improving diagnosis and treatment outcomes.

## 8. Technical Implementation of CDP-Based Interactome Models

### 8.1. Data Collection Protocols

Implementing CDP-based interactome analysis requires revised data collection protocols that emphasize and characterize variability rather than minimize it.

**Replicate Design:** Instead of averaging replicates to minimize noise, CDP approaches focus on preserving and analyzing variability between replicates. This requires careful experimental design to differentiate between technical and biological sources of variability. It is essential to include appropriate controls and standards to distinguish between measurement noise and genuine biological variability [155]. Multiple biological replicates should be processed independently throughout the entire experimental pipeline to capture inter-individual or inter-sample variability accurately. Technical replicates can be used to estimate measurement noise, allowing researchers to distinguish it from biological variability in subsequent analyses.

**Temporal Sampling:** Experiments designed to observe changes over time must have sufficient temporal resolution to accurately capture dynamic interaction networks. The required temporal resolution varies depending on the biological processes being investigated, ranging from seconds for rapid signaling events to days for developmental processes [240]. Sampling strategies should consider known biological rhythms, such as circadian cycles and the cell cycle, and include enough time points to capture both transient and sustained changes [241]. Adaptive sampling approaches that increase temporal resolution at critical transition points can enhance the amount of information gathered while minimizing the experimental burden.

**Condition Diversity:** A systematic exploration of various cellular conditions aims to map context-dependent interaction patterns. This exploration may involve different growth conditions, stress treatments, developmental stages, or disease states [242]. The objective is to characterize how interaction networks reconfigure in response to these varying contexts. Factorial experimental designs can efficiently examine multiple conditions at once, while sequential designs can adaptively focus on the most informative conditions [243]. High-throughput screening methods can systematically perturb cellular systems to investigate network variability [244].

**Single-Cell Resolution:** When possible, measurements are conducted at the single-cell level to capture intercellular variability [245]. This is especially important in heterogeneous systems, such as tumors, developing tissues, or mixed cell populations. Employing single-cell approaches requires careful attention to cell isolation methods, processing protocols, and analytical pipelines. These should be designed to preserve biological variability while minimizing technical artifacts [246,247].

### 8.2. Statistical Analysis Methods

Analyzing data based on the CDP requires specialized statistical methods that can effectively model and interpret variability.

**Variance Modeling:** Explicit modeling of variance components helps differentiate between various sources of variability [248]. Hierarchical variance models can separate these sources into inter-individual, inter-cellular, and technical factors. This approach enables the identification of the biological factors that drive variability in interactions. Mixed-effects models can incorporate both fixed effects, which represent systematic differences between conditions, and random effects, which account for variability around the average effects [249]. Variance component analysis quantifies the relative contributions of these different sources of variability [250,251,252].

**Hierarchical Models:** Multi-level models are designed to account for variability across different biological scales, including molecular, cellular, tissue, and organismal levels [253]. These models can illustrate how variability at one level affects variability at other levels, offering insights into the propagation of biological noise throughout biological systems. Bayesian hierarchical models are especially suitable for this purpose, as they can effectively integrate prior knowledge about biological systems while learning from data [254]. Additionally, these models effectively propagate uncertainty across hierarchical levels [255].

**Time Series Analysis:** Methods for analyzing temporal dynamics in interaction data involve both parametric and nonparametric approaches. Parametric methods include differential equation models, while non-parametric methods include Gaussian process regression. The choice of method depends on the specific characteristics of the temporal dynamics and the available data [256]. State-space models are useful for capturing hidden states that influence observed dynamics, whereas vector autoregressive models can help identify temporal dependencies among different components of a network [257]. Additionally, machine learning techniques such as recurrent neural networks are effective at recognizing complex temporal patterns in high-dimensional data [258].

**Clustering and classification techniques:** These are essential for identifying distinct patterns of variability and grouping similar network states. Clustering algorithms need to be adapted to handle high-dimensional, noisy data while preserving biologically relevant patterns [259]. Mixture model approaches can help identify distinct subpopulations with different interaction patterns [260]. Non-parametric clustering methods, such as density-based clustering, can effectively recognize clusters with complex shapes and varying densities [261]. Additionally, consensus clustering can provide robust cluster assignments by integrating multiple clustering algorithms [262,263].

### 8.3. Network Representation

CDP-based interactome models need innovative methods for network representation that can effectively capture variability and uncertainty [91,92,93].

**Weighted Edges:** Representing interaction strength with continuous weights rather than binary connections allows for a more nuanced understanding of relationships. Edge weights can reflect interaction probabilities, binding affinities, or functional coupling strengths. These weights should be allowed to vary across different conditions and time points [264,265]. Additionally, uncertainty in edge weights should be explicitly represented using confidence intervals or probability distributions. This approach enables the propagation of uncertainty through network analyses and aids in identifying the most reliable features of the network [266,267].

**Temporal Networks:** Network representations that explicitly incorporate temporal information can be created in various ways. This may include using time-varying edge weights, capturing discrete snapshots of the network at different time points, or employing continuous-time network models [268]. Dynamic network visualization tools can help researchers understand how networks evolve. Through animation and interactive visualization techniques, temporal patterns can be revealed that might otherwise be overlooked in static representations [269,270].

**Uncertainty Quantification:** Visual and analytical methods are employed to represent uncertainty in network structures, encompassing both the existence of edges and their weights [271]. Visualizing uncertainty is challenging but crucial for accurately interpreting network models. Ensemble approaches that depict networks as collections of possible structures can effectively capture this structural uncertainty. Additionally, bootstrap sampling and Bayesian methods can provide estimates of uncertainty for network parameters [272,273].

**Multi-Layer Networks:** Different types of interactions, such as protein-protein, protein-DNA, and metabolic interactions, can be represented in a multi-layer network, which illustrates their interdependencies [274]. These networks capture the full complexity of cellular interaction systems, enabling the identification of regulatory relationships across different layers. Analyzing specific layers and their interactions can reveal various aspects of network organization. Additionally, multiplex network measures can quantify the interdependence across the different interaction layers [275,276].

## 9. Validation and Benchmarking

### 9.1. Validation Strategies

Validating interactome models based on CDPs necessitates comprehensive validation strategies that account for their probabilistic and dynamic nature.

**Predictive Accuracy:** Evaluating the capability of CDP models to predict cellular behavior in novel conditions involves dividing the data into training and test sets. This division must effectively assess the model’s ability to generalize to new contexts. Cross-validation strategies should consider both the temporal and contextual structure of the data [277]. For instance, temporal cross-validation should focus on predicting future time points, whereas contextual cross-validation should assess predictions under new experimental conditions [278,279].

**Mechanistic Validation:** Validation of key predictions through targeted perturbation studies is essential. Computational models of cellular dynamics should generate specific, testable predictions regarding how changes in network interactions will influence cellular behavior [280]. Tools such as CRISPR-based gene editing, optogenetic control, and pharmacological interventions can be used to test these model predictions. Additionally, it is essential to compare the variability of responses to perturbations with the models’ predictions.

**Independent Validation:** Validation should involve independent datasets generated by different laboratories or using various experimental platforms. This approach tests how well CDP models can generalize beyond the specific datasets from which they were developed [281]. Meta-analysis methods can be employed to combine results from multiple independent validation studies [282]. Additionally, systematic benchmarking initiatives could provide standardized datasets and evaluation metrics for comparing different modelling approaches.

**Functional Validation:** Evaluating whether CDP models capture biologically significant patterns involves several approaches. This may include comparing the models with established biological pathways, correlating them with functional genomics data, or assessing their consistency with evolutionary trends. Additionally, gene ontology enrichment analysis can determine if network modules align with recognized functional categories [283,284]. Furthermore, analyzing evolutionary conservation can reveal whether key features of the network are preserved across different species [285,286].

### 9.2. Benchmarking Approaches

Benchmarking CDP-based models against traditional approaches requires consideration of their differing assumptions and capabilities.

**Performance Metrics:** Developing metrics that effectively evaluate dynamic, probabilistic network models is essential [287,288]. Traditional metrics, designed for static, binary networks, may not be suitable for CDP-based models. Area under the curve (AUC) metrics can be used to assess the performance of binary classification. Mean-squared error and similar metrics can be used to evaluate continuous predictions. Additionally, specialized metrics may be necessary to accurately assess temporal prediction performance.

**Baseline Comparisons:** A systematic comparison of existing interactome construction methods across multiple datasets and evaluation criteria is essential [289]. This comparison should encompass both traditional experimental approaches and computational prediction methods. To ensure a fair assessment, it is necessary to carefully consider the different data requirements and assumptions associated with each method [290]. While some traditional methods may excel at specific tasks, they may not perform as well when modeling variability is required [291,292].

**Biological Relevance:** Evaluating the effectiveness of various approaches in capturing known biological phenomena is essential. This evaluation may involve assessing the ability to replicate known regulatory relationships, predicting genes associated with diseases, or maintaining consistency with functional studies [293]. Systematic literature mining can uncover well-established biological relationships that can serve as gold standards. Additionally, expert curation by biological specialists can offer further validation data [294,295].

**Computational Efficiency:** Evaluation of the computational requirements of various approaches reveals that CDP-based models are inherently more complex than traditional methods. Therefore, any efficiency comparisons should consider the additional information provided by these models. Scalability analysis must be conducted to assess how different approaches perform as the network size increases. It is also essential to characterize memory and runtime requirements for various types of analyses [296,297].

## 10. Future Directions and Challenges

### 10.1. Technical Challenges

To fully harness the potential of CDP-based interactomics, several technical challenges need to be addressed.

**Computational Complexity:** CDP models are more complex than traditional methods, requiring advanced computational techniques and resources. Their probabilistic nature and the need to account for multiple sources of variability significantly increase computational demands. To address these challenges, efficient algorithms for probabilistic network inference, parallel computing methods for large-scale analyses, and approximate approaches for solving computationally intensive problems will be necessary.

**Data Requirements:** CDP approaches necessitate larger and more diverse datasets than traditional methods, presenting challenges for data generation, storage, and management. Capturing variability across multiple conditions, time points, and biological contexts significantly increases the data requirements. To facilitate data sharing and integration, standardized data formats and metadata standards will be essential. Additionally, automated experimental platforms and high-throughput methods will be necessary to generate the required data volumes.

**Parameter Estimation:** Estimating the parameters of complex, dynamic network models using limited data presents significant challenges. The high dimensionality of biological networks, combined with the complexity of CDP models, complicates the parameter estimation process. To address these challenges, regularization methods, incorporation of prior information, and transfer learning approaches can be beneficial. Additionally, employing an adaptive experimental design, which strategically selects experimental conditions to maximize the information gained, could enhance the efficiency of parameter estimation [298].

**Model Selection:** Selecting the appropriate model complexity and structure across diverse biological systems can be a challenging task. CDP models provide numerous modeling options, making it essential to thoughtfully choose the most suitable approach for a specific biological question. To aid this selection process, model comparison methods that assess both the quality of the fit and the model’s complexity are necessary. Additionally, cross-validation techniques tailored for complex, dynamic models could assist in selecting the most appropriate model.

### 10.2. Methodological Developments

Several methodological advancements could improve CDP-based interactomics.

**Improved Experimental Methods:** The development of new experimental techniques aims to capture molecular interaction dynamics with increased precision, resolution, and throughput. This encompasses advancements in mass spectrometry, fluorescence microscopy, and single-cell technologies. Proximity labeling methods are effective at capturing transient or weak interactions that traditional methods often overlook. Additionally, super-resolution microscopy offers spatial information about the locations of interactions within cells. Microfluidic devices enable precise control over cellular environments while facilitating high-throughput measurements [299].

**Advanced Modeling Approaches:** Developing more advanced mathematical models is essential for accurately representing the complexity of biological variability. This may involve using nonlinear dynamical models, machine learning techniques, and hybrid modeling strategies. Physics-informed neural networks could integrate established biological constraints while learning intricate patterns from data. Additionally, generative models could simulate realistic biological variability, which would aid hypothesis testing and experimental design.

**Integration Methods:** Developing methods to integrate various omics data types within the CDP framework is essential. Multi-omics integration poses specific challenges due to the differing noise characteristics and biological interpretations associated with each data type. Utilizing graph-based integration methods can effectively represent relationships between different molecular layers. Additionally, tensor factorization approaches can help identify shared patterns across multiple data types and conditions [300].

**Causal Inference:** Methods for inferring causal relationships in complex, dynamic systems are crucial. Traditional correlation-based approaches do not adequately capture the causal structure of biological networks. By combining experimental perturbation data with causal inference algorithms, we can identify causal relationships within these networks. Additionally, adapting instrumental variable techniques to biological systems may help distinguish correlation from causation.

### 10.3. Applications and Impact

Successfully implementing CDP-based interactomics could have significant effects on both biology and medicine.

**Personalized Medicine:** More accurate and individualized interaction models could facilitate genuinely personalized therapeutic approaches. By considering individual differences in network structure and dynamics, therapies can be tailored to each patient [301]. Applications of pharmacogenomics may enable the prediction of individual drug responses using these network models. Additionally, discovering biomarkers could help identify network-based signatures that predict treatment outcomes.

**Drug Discovery:** A deeper understanding of target networks and their inherent variability could significantly enhance the drug discovery and development process. CDP-based models can be utilized to identify new drug targets, predict potential side effects, and assist in the design of combination therapies. Network-based drug repositioning may reveal new applications for existing medications. Additionally, resistance prediction models can help develop treatments that are less susceptible to failure due to evolving resistance.

**Systems Biology:** CDP-based approaches have the potential to enhance our understanding of biological systems and their emergent properties. This could lead to new theoretical insights into the principles of biological organization. Applications in synthetic biology could benefit from a better understanding of how to engineer robust and controllable biological systems. In addition, biotechnology applications could leverage biological variability to enhance production systems.

## 11. Conclusions

The Constrained Disorder Principle (CDP) represents a significant shift in how we study the interaction networks of molecular, cellular, and whole organs. Instead of trying to eliminate biological variability and noise, the CDP embraces these elements, offering a pathway to create more accurate, biologically relevant interactome models that reflect the dynamic and stochastic nature of living systems. It considers variability essential for preserving the system’s integrity.

This comprehensive analysis reveals that variability is not a barrier to understanding biological systems; instead, it is a fundamental characteristic that plays essential functional roles. From molecular-level processes such as stochastic gene expression and protein conformational dynamics to system-level phenomena like immune system diversity, heart rate variability, neuronal variability, and gait variability, controlled variability enables capabilities that would be unattainable in purely deterministic systems. The evidence presented spans multiple scales of biological organization, demonstrating consistent patterns: biological systems have evolved to utilize variability as a functional resource while maintaining appropriate constraints to ensure viability and performance. This suggests that the CDP captures a core principle of biological organization.

Advancing the implementation of the CDP in interactome research will require significant improvements in both experimental and computational methodologies, as well as the use of more comprehensive datasets. However, the potential benefits are substantial, including improved predictive accuracy of cellular behavior, better disease models that account for patient heterogeneity, and more effective therapeutic strategies that consider network variability and dynamics.

The case studies examined illustrate how CDP-based approaches may provide new insights into complex biological phenomena. In cancer research, accounting for tumor heterogeneity and network dynamics could lead to therapies that prevent or delay the evolution of resistance. In neurodegenerative diseases, understanding how pathology disrupts network variability could guide earlier interventions and enable personalized treatments. In immunology, modeling immune system variability could facilitate the development of more effective vaccines and treatments for autoimmune diseases.

The technical implementation of CDP-based interactomics may present both challenges and opportunities. Advanced experimental techniques, such as single-cell technologies and time-resolved measurements, are increasingly enabling the capture of the variability that CDP models require. Additionally, computational advances in machine learning, probabilistic modeling, and high-performance computing are providing the tools necessary to analyze complex, high-dimensional data.

Integrating the CDP into interactome research signifies a technical advancement and a conceptual shift that recognizes variability as an inherent characteristic of biological systems, rather than a flaw. This shift in perspective has the potential to transform our understanding of life and open new avenues for scientific discovery and medical application. The implications extend beyond interactome research to the broader field of systems biology. The CDP may provide a framework for understanding how complex systems can be both robust and flexible, stable and adaptive. These insights could inform the design of artificial systems that exhibit similar properties, ranging from robust engineered systems to adaptive artificial intelligence. Understanding that controlled variability is essential for life challenges us to rethink many aspects of biological research, including experimental design, theoretical modeling, and therapeutic interventions. Future research will demonstrate whether implementing CDP-based interactomes can enhance the accuracy of modeling physiological systems.

The CDP provides a unifying framework for potentially improved understanding of the seemingly paradoxical nature of biological systems: how they can be both precise enough to maintain essential functions and flexible enough to adapt to changing conditions. The answer lies in the sophisticated balance between order and disorder. The future of interactome research depends not on eliminating variability but on its principled incorporation into our models and understanding.

## Data Availability

No new data were created or analyzed in this study.
